# Dismantling Psychological Interventions for Youth with Complex Trauma: A Systematic Review and Component Network Meta-Analysis

**DOI:** 10.1159/pps/aemag009

**Published:** 2026-10-10

**Authors:** Yu Li, Junkai Feng, Wanyan Hu, Zhuo Zeng, Qian Pan, Jun Wang, Muyang Li, Thomas Elbert, Anke Karl, Yinyin Zang

**Affiliations:** a School of Psychological and Cognitive Sciences, Peking University, Key Laboratory of Machine Perception (Ministry of Education), and Beijing Key Laboratory of Brain-Computer Interface and Mental Health Modulation, Beijing, China; b School of Health Humanities, Peking University, Beijing, China; c Department of Psychology, University of Konstanz, Konstanz, Germany; d Department of Psychology, University of Exeter, Exeter, UK

**Keywords:** Component network meta-analysis, Adolescents, Complex trauma, Posttraumatic stress disorder, Psychological interventions

## Abstract

**Introduction:**

Complex trauma-related posttraumatic stress disorder (PTSD) and comorbidities pose a significant mental health burden in children. However, the comparative effectiveness of psychological interventions and shared therapeutic components remains unclear.

**Methods:**

We conducted a systematic review, network, and component network meta-analysis of randomized controlled trials of psychological interventions for children and adolescents with complex trauma. PubMed, Embase, Web of Science, PsycINFO, and Cochrane Library were searched up to September 2024. PTSD was the primary outcome; retention and comorbid symptoms (depression, anxiety, dissociation, externalizing problems) were secondary outcomes.

**Results:**

Forty-eight studies (6,017 participants) were included in this review. Multimodal group interventions (SMD −1.68, 95% confidence interval [CI] −3.04 to −0.32), phase-based trauma-focused cognitive behavioral therapy (TF-CBT) with parental involvement (−1.12, −1.53 to −0.70), TF-CBT (−1.06, −1.59 to −0.52), non-TF-CBT with parental involvement (−0.93, −1.70 to −0.17), eye movement desensitization and reprocessing (−0.85, −1.60 to −0.10), and phase-based TF-CBT (−0.79, −1.20 to −0.39) significantly reduced PTSD. Exposure emerged as the only significantly effective component for PTSD (−0.85, 95% credible interval [CrI] −1.60 to −0.06). Efficient component combinations included affective modulation with a therapeutic foundation of psychoeducation and nonspecific elements for PTSD (−0.97, 95% CrI −1.92 to −0.01) and exposure plus parental involvement with the same foundation for depression (−2.11, 95% CrI −4.06 to −0.20).

**Conclusion:**

Trauma-focused approaches were the most effective treatments for pediatric PTSD and emotional comorbidities related to complex trauma. A more cost-effective intervention approach may prioritize exposure techniques, incorporate affective modulation, and include parental involvement when feasible.

## Introduction

Complex trauma in children and adolescents constitutes a global health emergency with devastating mental health consequences. Unlike single-incident trauma, complex trauma, arising from prolonged exposure to severe adversity such as abuse, war, or forced displacement [1], fundamentally disrupts development and costs the USA alone USD 124 billion from 1 year’s new cases [2]. Recent meta-analytic evidence indicates that 12–20% of trauma-exposed children and adolescents develop posttraumatic stress disorder (PTSD), with prevalence substantially higher among those exposed to interpersonal trauma [3]. Such exposure is also associated with elevated risks of emotional dysregulation, dissociation, behavioral problems, and persistent psychiatric morbidity into adulthood [4, 5].

Epidemiological evidence underscores the magnitude of this public health challenge. Nearly half of adolescent trauma cases are complex [6–8], reaching 77.6% in adolescent psychiatric populations [9]. The problem is intensifying, and the global displaced population (40% children) has nearly doubled to 122.6 million since 2015 [10], while technology-facilitated abuse has increased child sexual victimization from 13.5% to 21.7% [11]. Despite this burden, only one-fifth of affected children receive any professional mental health support, even in high-income countries [12, 13]. This treatment gap widens dramatically in low-resource settings [14], creating a situation where service deficiencies are most pronounced precisely where the need is greatest, underscoring the urgency of scalable, evidence-informed solutions.

There has been a long-standing concern that exposure to complex trauma is associated with more severe symptomatology and poorer treatment outcomes [15–17]. Prior reviews have established the efficacy of trauma-focused interventions such as trauma-focused cognitive behavioral therapy (TF-CBT) and eye movement desensitization and reprocessing (EMDR) for pediatric PTSD. However, heterogeneity in inclusion criteria, particularly regarding trauma type and exposure characteristics, has contributed to variation in treatment effect estimates and recommendations [18–20]. Uncertainty remains regarding optimal treatment strategies for youths exposed to complex trauma. For example, although phase-based approaches emphasizing initial stabilization have long been recommended [21], recent randomized trials [22–25] indicate no added benefit over direct trauma-focused interventions, raising concerns about potentially unnecessary delays in care [26]. Moreover, given that many psychotherapies share overlapping components [27], identifying the specific elements that drive treatment effects is critical for developing scalable and resource-efficient interventions in the context of global therapist shortages and constrained healthcare resources [28, 29]. In addition, with the introduction of complex PTSD (CPTSD) as a diagnosis distinct from PTSD in ICD-11, this issue has gained renewed relevance. Although complex trauma exposure and CPTSD are conceptually distinct and should not be used interchangeably, CPTSD is more prevalent among individuals with histories of repeated or prolonged trauma [30, 31]. Given the current scarcity of CPTSD-focused intervention studies, synthesizing evidence from samples defined by complex trauma exposure may provide clinically relevant insights and inform future intervention research on CPTSD [1, 32].

To address these critical gaps, we conducted a comprehensive meta-analytic synthesis of psychological interventions for adolescents experiencing complex trauma. While previous reviews have largely focused on PTSD, depression, and anxiety [33–35], our study extends this scope by also examining dissociation and externalizing problems, which are frequently observed in clinical settings but less often evaluated in trials. Using a multilevel meta-analytic approach, including pairwise meta-analysis, network meta-analysis (NMA), and component NMA (CNMA), we assessed both the comparative effectiveness of interventions and the active therapeutic components driving improvement. We also examined treatment retention rates across interventions, the relative impact of phase-based versus non-phase-based approaches, and applied a novel analytic method to identify the minimal sufficient combination of components required for optimal outcomes. Our findings aim to inform clinical guidelines and health policy, improving access to effective and resource-efficient interventions for these high-risk populations globally.

## Methods

This systematic review and meta-analysis were prospectively registered (CRD42024522444) and can be accessed at https://www.crd.york.ac.uk/PROSPERO/view/CRD42024522444. The report was developed in accordance with the Preferred Reporting Items for Systematic Reviews and Meta-Analyses (PRISMA) guidelines, as well as the PRISMA extension for NMAs [36, 37]. The completed checklist is provided in online supplementary material 16 (for all online suppl. material, see https://doi.org/10.1159/pps/aemag009).

### Search Strategy

We conducted a systematic search of PubMed, Embase, Web of Science, PsycINFO, and Cochrane Library from database inception to September 20, 2024, to identify randomized controlled trials (RCTs) published in English or Chinese. The detailed search strategy is provided in online supplementary material 1. Additional studies were identified by screening the reference lists of related systematic reviews published between January 1, 2021, and September 20, 2024.

### Eligibility Criteria

We included RCTs evaluating psychological interventions meeting the following criteria: (1) conducted in children or adolescents (<19 years old); (2) participants exposed to complex trauma, defined as traumatic experiences meeting at least one of the following criteria [1, 38, 39]: prolonged or cumulative exposure (e.g., multiple traumatic events, war, or armed conflict), severe interpersonal threat (e.g., physical or sexual abuse), or substantial disruption to the caregiving environment (e.g., forced displacement, domestic violence, or parental death); (3) PTSD symptom severity as the primary outcome; and (4) peer-reviewed publications. For trials with multiple reports, we selected the publication with the longest follow-up period.

The following intervention categories were included: (1) TF-CBT, comprising CBT-based interventions with explicit trauma-processing components (e.g., prolonged exposure, cognitive processing therapy); (2) EMDR; (3) non-trauma-focused CBT, including CBT-based approaches without direct trauma processing (e.g., emotion regulation training, interpersonal skills training); (4) supportive or nondirective counselling; (5) psychodynamic therapies; (6) third-wave therapies (e.g., mindfulness-based approaches); (7) child-specialized interventions, comprising developmentally adapted approaches (e.g., play therapy, child-parent psychotherapy); (8) multicomponent interventions integrating multiple therapeutic modalities. Interventions lacking a recognized psychological foundation (e.g., neurofeedback, yoga) were excluded. The following comparators were also included: (1) passive control conditions, such as waiting list or no-treatment controls; or (2) active control conditions, such as treatment as usual, psychological placebo, supportive assessment, or other minimal-contact controls.

### Outcomes

The primary outcome was the change in PTSD symptom severity from baseline to posttreatment, short-term follow-up (≤6 months), and long-term follow-up (>6 months), assessed using standardized scales or structured interviews. Secondary outcomes included treatment retention, defined as completion of the final treatment session post-randomization, and reductions in comorbid symptoms, including depression, anxiety, dissociation, and behavioral problems, assessed using standardized measures. Effect sizes were reported as standardized mean differences (SMDs) for continuous outcomes and odds ratios (ORs) for dichotomous outcomes.

### Data Extraction and Quality Assessment

After removing duplicates, pairs of independent reviewers (Y.L., W.H., Z.Z., Q.P., and J.W.) screened titles and abstracts, followed by full-text review to identify eligible studies. Two reviewers independently extracted data using a structured spreadsheet, recording key characteristics such as participant demographics, setting, intervention details, attrition rates, and outcome measures. Intention-to-treat data were prioritized over complete-case data when available. To ensure consistency, self-reported outcomes were preferred over clinician- or observer-reported assessments. Discrepancies were resolved through discussion. Risk of bias was assessed independently by two reviewers (Y.L. and J.W.) using the Cochrane Risk of Bias 2.0 (RoB 2) tool [40].

Intervention categories for NMA and treatment component definitions for CNMA were developed using predefined frameworks informed by existing PTSD treatment guidelines and relevant literature. Both frameworks were specified a priori and refined through an expert consensus process involving a registered doctoral-level clinical psychologist (Y.Z.) and two doctoral-level researchers (Y.L. and J.F.). Two independent reviewers (Y.L. and J.F.) applied the frameworks to all included interventions, resolving discrepancies through discussion, with arbitration by the clinical psychologist when necessary. Inter-rater reliability was quantified using Cohen’s κ. Definitions of intervention categories and study-by-study classification are provided in online supplementary material 12.

### Statistical Analyses

We conducted pairwise meta-analyses comparing psychological interventions with control conditions (inactive controls such as waitlists, or active controls such as treatment-as-usual), excluding head-to-head comparisons of active interventions. Subgroup analyses were performed by treatment type and treatment setting to explore potential sources of heterogeneity. Proportion meta-analyses estimated dropout rates across intervention arms and subgroups.

An NMA was then performed to compare relative effectiveness across interventions. To assess transitivity, we examined distributions of potential effect modifiers, including treatment setting, proportion of female participants, publication year, mean age, and sample size. The frequentist approach implemented in the R package “netmeta” [41] was used, assuming a common heterogeneity parameter across comparisons [42]. Between-study variance (τ^2^) was calculated and reported for each outcome. Network inconsistency was evaluated using the design-by-treatment interaction model [43] and back-calculation methods [44] to separate direct and indirect evidence.

To refine comparisons at the component level, we conducted a CNMA, assuming that intervention effects result from the sum of individual components without interactions [45, 46]. Components were coded as binary presence/absence indicators and therefore did not capture implementation differences, such as intensity or sequencing. Further details are provided in online supplementary material 13. Models were fitted using the R package “rjags,” an interface for JAGS (Just Another Gibbs Sampler), enabling Bayesian estimation via Markov Chain Monte Carlo methods [47]. Sensitivity analyses tested the robustness of the additive component-effects assumption, incorporating age, sex proportion, and pretest standardized scores as covariates. Model fit was assessed using the deviance information criterion, and convergence was evaluated via trace plots and the Brooks-Gelman-Rubin statistic; all models achieved convergence after 25,000 iterations.

To identify optimal therapeutic configurations, we systematically added treatment components in descending order of effect sizes to a two-component baseline comprising nonspecific therapeutic elements and psychoeducation. Nonspecific elements, such as therapeutic alliance, companionship, and empathic engagement, were assumed to be present across all interventions. Psychoeducation was explicitly reported in most protocols. From this foundation, we determined the minimal sufficient component combination required to achieve statistically significant outcomes. This sequential additive approach enabled the identification of parsimonious therapeutic frameworks with maximal clinical utility.

Potential reporting bias was assessed using visual inspection of funnel plots and Egger’s regression test to examine small-study effects in pairwise meta-analyses comparing active interventions with control conditions. Further sensitivity analyses were conducted, excluding studies with a high overall risk of bias.

## Results

### Study Selection and Characteristics

Our systematic search identified 4,832 non-duplicate references, from which 206 full-text articles were reviewed. Of these, 48 studies met the eligibility criteria for systematic review, and 40 studies provided data for the meta-analysis (Fig. 1). Details of included studies and excluded studies are shown in online supplementary material 2.

**Fig. 1.**
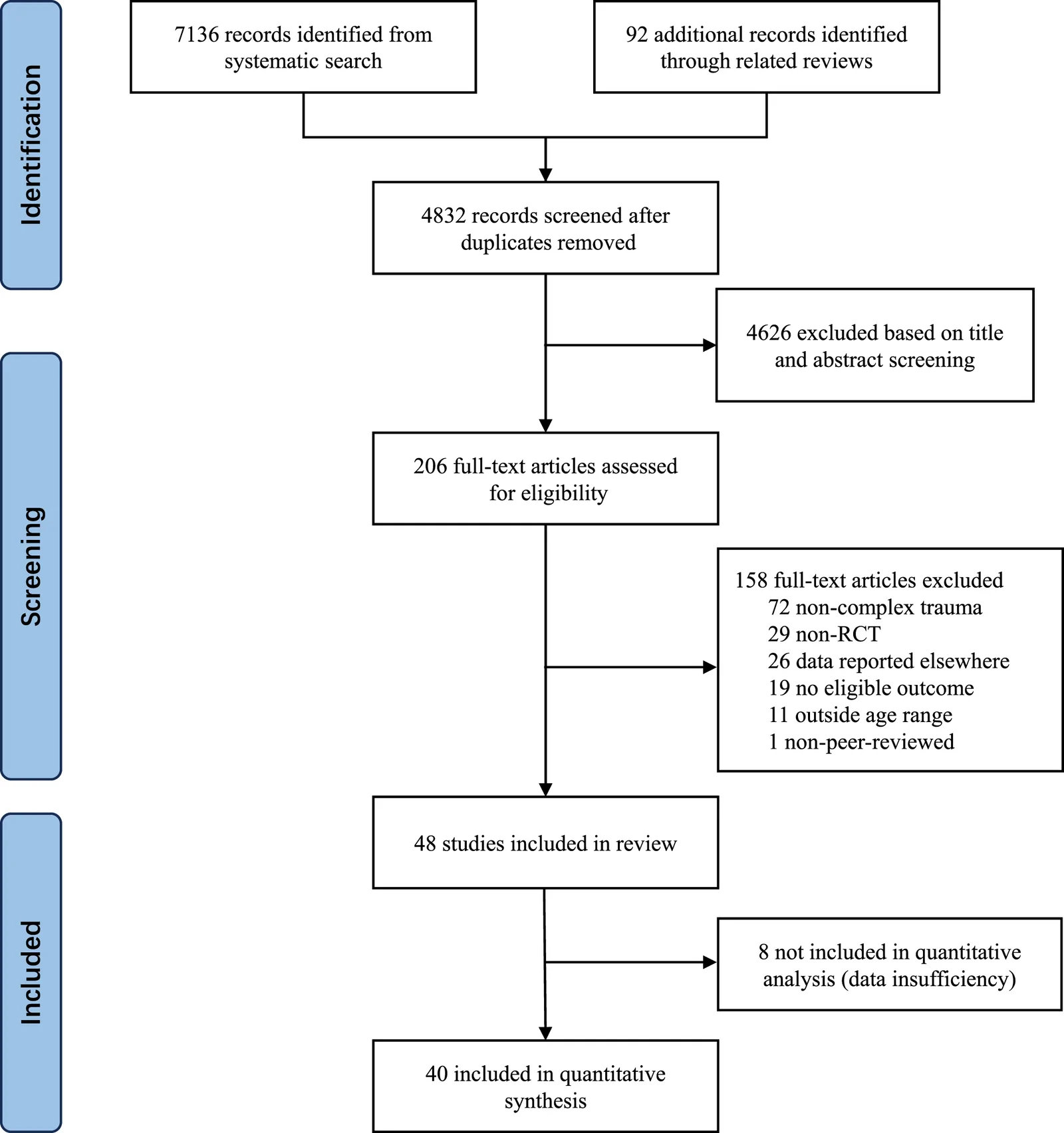
Flowchart of the selection of studies.

The 48 included studies involved 6,017 children and adolescents (mean age: 11.7 years, range: 2–18.8 years), with 52% female participants. Twenty studies (42%) involving 3,428 participants (57%) were conducted in low- and middle-income countries. Treatment duration ranged from 5 to 50 sessions (mean: 13.25). The most common trauma exposure was war or armed conflict (40%), followed by maltreatment (23%), bereavement (11%), refugee displacement (11%), domestic violence (9%), and multiple or prolonged trauma (7%). TF-CBT was the most frequently evaluated intervention (35 studies, 73%), with 19 group-based arms and 22 phase-based approaches. Parental involvement was reported in 17 studies (35%). Most interventions were delivered by professional therapists (e.g., psychologists, licensed therapists, experienced counselors; *n* = 34, 71%), while 14 studies (29%) used paraprofessionals (e.g., trained social workers, teachers, or graduate students). Follow-up assessments were conducted within 6 months in 25 studies (52%) and beyond 6 months in 9 studies (19%). Full characteristics of the included studies are shown in online supplementary material 3.

### Risk of Bias

Among the 40 studies included in the meta-analyses, 21 (52%) had a moderate risk of bias, 14 (35%) had high risk, and 5 (13%) had low risk. The most common sources of bias were the lack of allocation concealment and the absence of a prespecified data analysis plan, both of which could impact the reliability of the results. Full risk-of-bias assessments are provided in online supplementary material 4.

### Pairwise Meta-Analyses

Psychological treatments significantly reduced PTSD symptoms at posttreatment (41 trials; SMD −0.65 [95% CI: −0.84 to −0.45]; *I*^2^ = 84%), short-term follow-up within 6 months (21 trials; −0.60 [−0.88 to −0.32]; *I*^2^ = 82%), and long-term follow-up beyond 6 months (7 trials; −0.44 [−0.57 to −0.32]; *I*^2^ = 0%). Depression symptoms were also significantly reduced at posttreatment (29 trials; −0.51 [−0.75 to −0.27]; *I*^2^ = 83%), short-term follow-up (16 trials; −0.38 [−0.57 to −0.18]; *I*^2^ = 62%), and long-term follow-up (6 trials; −0.39 [−0.52 to −0.26]; *I*^2^ = 0%). Anxiety symptoms showed significant reductions at posttreatment (11 trials; −0.34 [−0.58 to −0.10]; *I*^2^ = 69%) and short-term follow-up (7 trials; −0.19 [−0.33 to −0.05]; *I*^2^ = 5%), though long-term data were insufficient to conduct the analysis. In contrast, no significant reductions were found for externalizing problems (8 trials; −0.06 [−0.19 to 0.07]; *I*^2^ = 5%) or dissociation symptoms (3 trials; −0.16 [−0.38 to 0.07]; *I*^2^ = 0%). Retention rates across studies ranged from 0.62 to 1.00. The pooled retention rate at posttreatment was high (47 trials; 0.92 [0.88–0.95]; *I*^2^ = 61%) and had no significant difference with inactive controls (41 trials; OR 0.83 [0.56–1.19]; *I*^2^ = 53.5%). Retention rates by treatment type were as follows: phase-based TF-CBT with parental involvement (P+PB-CBT, 14 trials; 0.88 [0.80–0.93]); TF-CBT (7 trials; 0.88 [0.80–0.93]); phase-based TF-CBT (PB-CBT, 8 trials; 0.94 [0.88–0.97]); non-TF-CBT (4 trials; 0.81 [0.66–0.91]); child-parent psychotherapy (CPP, 1 trial; 0.86 [0.71–0.95]); EMDR (2 trials; 0.98 [0.06–1.00]); community- or classroom-based intervention (CBI, 4 trials; 0.99 [0.69–1.00]); multimodal group intervention (MGI, 1 trial; 0.86 [0.65–0.97]); expressive group (EG, 1 trial; 0.86 [0.77–0.92]); child-centered play therapy (CCPT, 1 trial; 1.00 [0.77–1.00]); parent intervention (P, 2 trials; 0.80 [0.68–0.88]); non-TF-CBT with parental involvement (P+non-TF-CBT, 2 trials; 1.00 [0.01–1.00]). Subgroup analyses indicated that neither trauma type (maltreatment, multiple or prolonged trauma, war or armed conflict, bereavement, refugee displacement, domestic violence) nor treatment setting (outpatient, community and social service settings, school) significantly influenced PTSD treatment effects. Comprehensive details of treatment effects across different interventions, comparison conditions, and time points are provided in online supplementary material 5. In the absence of publication bias or small-study effects, funnel plots are expected to show studies symmetrically distributed around the pooled effect estimate. The observed asymmetry suggested small-study effects, which were statistically supported by Egger’s test (*p* < 0.001). Smaller studies showed more variable and occasionally more extreme effect estimates than larger studies, a pattern that may reflect publication bias, between-study heterogeneity, or other methodological differences (online suppl. material 7).

### Network Meta-Analysis

NMA evaluated the effectiveness of psychological interventions for PTSD and depression at posttreatment. Data for other symptoms were insufficient for analysis. For PTSD, 39 studies contributed to the NMA. The network geometry is presented in Figure 2. Compared to inactive controls, several interventions significantly reduced PTSD symptoms, including MGI (−1.68 [−3.04 to −0.32]), P+PB-CBT (−1.12 [−1.53 to −0.70]), TF-CBT (−1.06 [−1.59 to −0.52]), P+Non-TF-CBT (−0.93 [−1.70 to −0.17]), EMDR (−0.85 [−1.60 to −0.10]), and PB-CBT (−0.79 [−1.20 to −0.39]). Despite varying effect sizes, no significant differences were found among these interventions. For depression, P+PB-CBT (−0.98 [−1.47 to −0.48]), PB-CBT (−0.72 [−1.18 to −0.26]), and TF-CBT (−0.63 [−1.19 to −0.07]) were significantly more effective than inactive control, without significant differences between these interventions. Regarding acceptability, ORs for treatment retention compared with inactive control ranged from 0.33 to 9.71, but differences between interventions were not significant. Detailed comparisons of therapy effectiveness and retention rates are available in Figure 3 and online supplementary material 8–11.

**Fig. 2.**
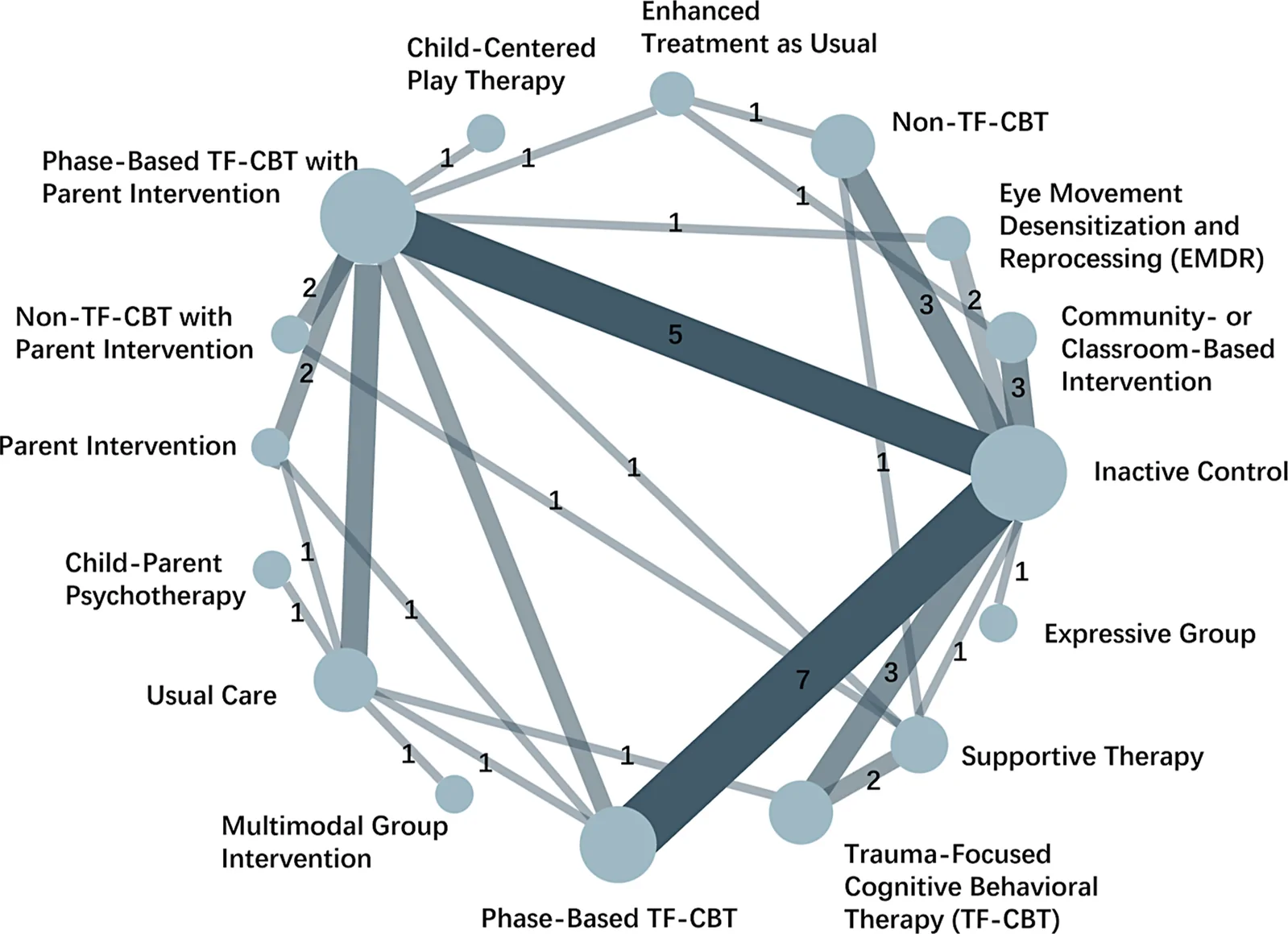
PTSD network diagram illustrating the number of studies per intervention category (node size) and the number of direct comparisons (line thickness, with numerical labels).

**Fig. 3.**
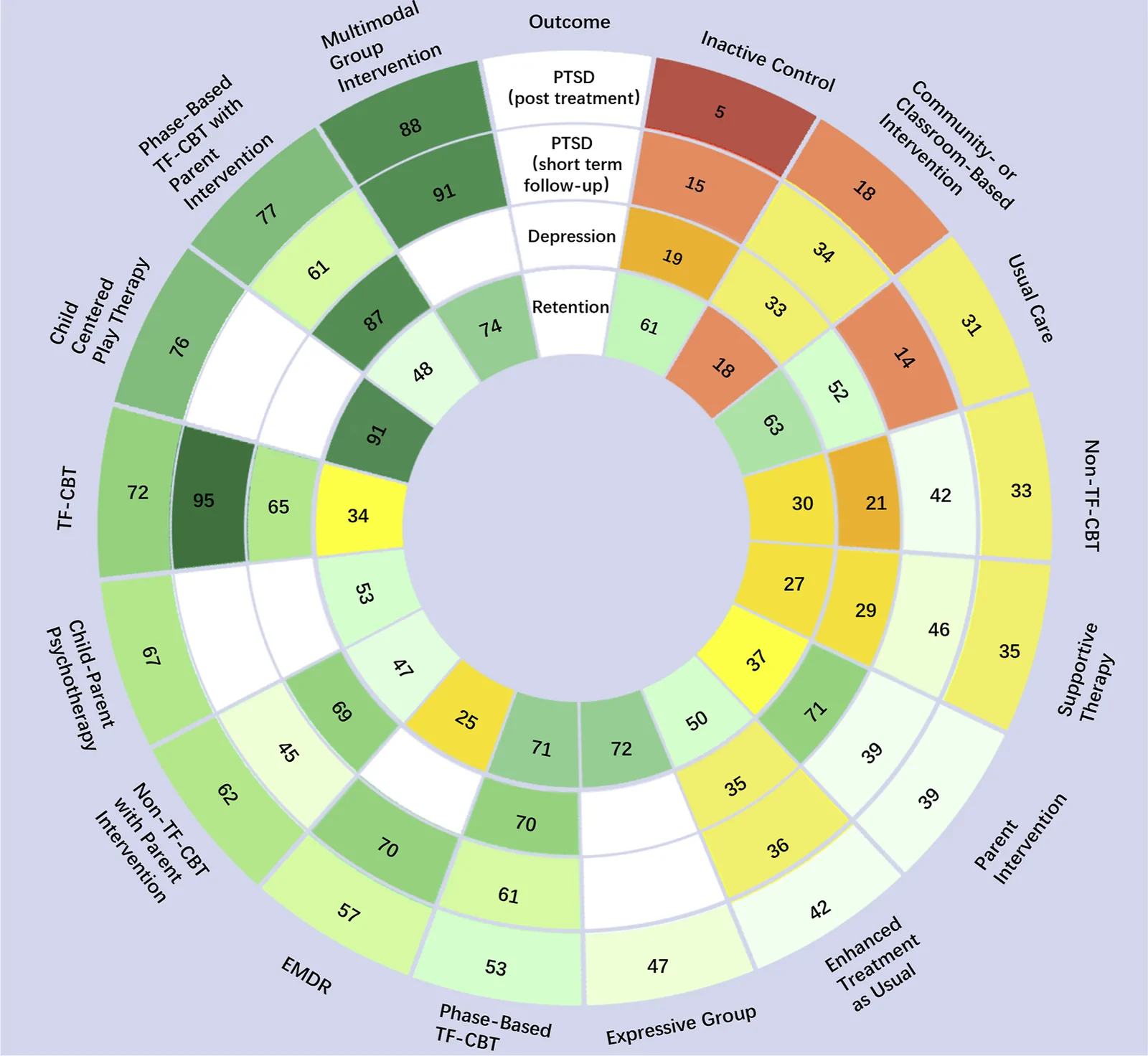
Rank-heat plot of therapies across different outcomes. This circular diagram presents the relative ranking of psychological interventions based on *p* scores (range 0–100) derived from the NMA across four outcomes: PTSD at posttreatment, PTSD at short-term follow-up, depression, and treatment retention. Each colored segment represents the probability that the corresponding intervention is superior to others for the specified outcome. Darker green shades indicate higher probabilities, reflecting greater effectiveness or retention. Interventions with more green segments demonstrate stronger or broader effectiveness across outcomes. White segments denote that the intervention was not included in the analysis for that outcome. TF-CBT, trauma-focused cognitive behavioral therapy; EMDR, eye movement desensitization and reprocessing; PTSD, post-traumatic stress disorder.

The distribution of potential effect modifiers (age, gender, treatment duration, and publication year) reasonably supported the transitivity assumption. However, the limited number of studies per comparison may have left some intransitivity undetected (online suppl. material 6). The estimated between-study variance indicated heterogeneity in the PTSD (τ^2^ = 0.26) and depression (τ^2^ = 0.28) networks; comparable magnitudes have been reported in previous meta-analyses [19]. This heterogeneity may reflect multiple study and intervention characteristics, including variation in treatment duration, delivery setting, comparator type, trauma exposure, and participant characteristics in complex-trauma populations. The design-by-treatment test for global inconsistency indicated significant global inconsistency in the NMA for PTSD (Q = 40.56, df = 14, *p* < 0.001) and depression (Q = 16.56, df = 8, *p* = 0.035). However, the back-calculation method found no evidence of inconsistency among treatment comparisons (online suppl. material 8–11).

### Component NMA

We extracted 18 treatment components from the included studies. Component definitions and the number of study arms per component are summarized in Table 1, with detailed study-by-component coding provided in online supplementary material 12. Inter-rater reliability was moderate to high across components (κ range = 0.67–0.92; online suppl. material 12). Without adjusting for covariates, exposure demonstrated a significant effect in reducing PTSD {SMD −0.85 (95% credible interval [CrI] −1.60 to −0.06)}. For depression, no single component had a significant effect, but parental involvement showed relatively larger effect estimates and a narrower posterior distribution (SMD −0.67 [95% CrI −1.55 to 0.23]). CNMA with covariates yielded consistent results.

**Table 1.** Definitions of components

Components	Description
Waiting list (22 arms)	A control condition where participants are monitored without receiving active treatment for a specified period, after which they receive the intervention
Creative-expressive exercises (17 arms)	Structured therapeutic use of creative modalities (visual arts, drama, dance, music) facilitating emotional expression and trauma processing through nonverbal channels guided by trained professionals
Cognitive restructuring (28 arms)	Systematic identification and modification of dysfunctional trauma-related thoughts and beliefs using techniques like Socratic questioning and cognitive reappraisal to develop more adaptive thinking patterns
Exposure (31 arms)	Systematic, therapist-guided confrontation of trauma memories or triggers through various modalities (e.g., imaginal, written, in vivo) with the goal of reducing avoidance and fear responses
Bilateral stimulation (11 arms)	EMDR-specific protocol using rhythmic bilateral sensory stimulation (visual, tactile, or auditory) while processing traumatic memories to facilitate adaptive information processing
Affective modulation (34 arms)	Structured training in identifying, understanding, and managing emotional responses, including specific skills for modulating the intensity and expression of emotions related to trauma
Group setting (27 arms)	Treatment delivered in a structured group format (specify size), facilitating therapeutic factors like normalization, peer support, and shared learning through structured interactions
Interpersonal skills (10 arms)	Structured training in social competencies, including communication, boundary-setting, and relationship-building, using techniques like role-play and behavioral rehearsal
3-wave technique (3 arms)	Integration of mindfulness, acceptance, and compassion-based approaches focusing on present-moment awareness and psychological flexibility rather than symptom elimination
Nonspecific treatment elements (58 arms)	Therapeutic benefits attributable to common factors across interventions (e.g., therapeutic alliance, expectancy effects) rather than specific technical components
Narrative (3 arms)	Structured development of coherent trauma narratives to integrate fragmented memories and experiences into meaningful personal stories, fostering meaning-making, emotional resilience, and control over their experiences
Usual care (9 arms)	Routine care practices available in the local healthcare system, including drug treatment, medical care, community services, and psychotherapy beyond specified therapeutic components
Parent involvement (28 arms)	Structured inclusion of caregivers in child/adolescent trauma treatment, including specific parent-focused interventions and joint parent-child sessions
Psychoeducation (44 arms)	Systematic provision of evidence-based information about trauma, PTSD, and recovery processes, including specific coping strategies and treatment rationale
Positive-oriented elements (12 arms)	Structured activities targeting strength development, resilience-building, and posttraumatic growth
Coping skills (30 arms)	Teaching skills address personal issues or strategies for recognizing and coping with potential traumas. For example, it includes instructing children who have experienced sexual abuse on how to recognize inappropriate physical contact and how to respond
Imagery work (6 arms)	Structured modification of trauma-related imagery using techniques like integrating safety elements (e.g., protective barriers, trusted companions) and creating new endings to reduce distress and build mastery. Unlike imagery exposure, which repeatedly confronts the original memory, imagery work actively alters its content
Relaxation (39 arms)	Systematic instruction in specific physiological arousal-reduction techniques (e.g., progressive muscle relaxation, controlled breathing) with regular practice components

In terms of the most parsimonious component combinations, adding affective modulation to the common therapeutic foundation (nonspecific treatment elements and psychoeducation) resulted in a significant reduction in PTSD symptoms (SMD −0.97 [95% CrI −1.92 to −0.01]). For depression, combining exposure and parental involvement with the common therapeutic foundation significantly reduced symptoms (SMD −2.11 [95% CrI −4.06 to −0.20]). Full results are presented in Figure 4 and online supplementary 13 and 14.

**Fig. 4.**
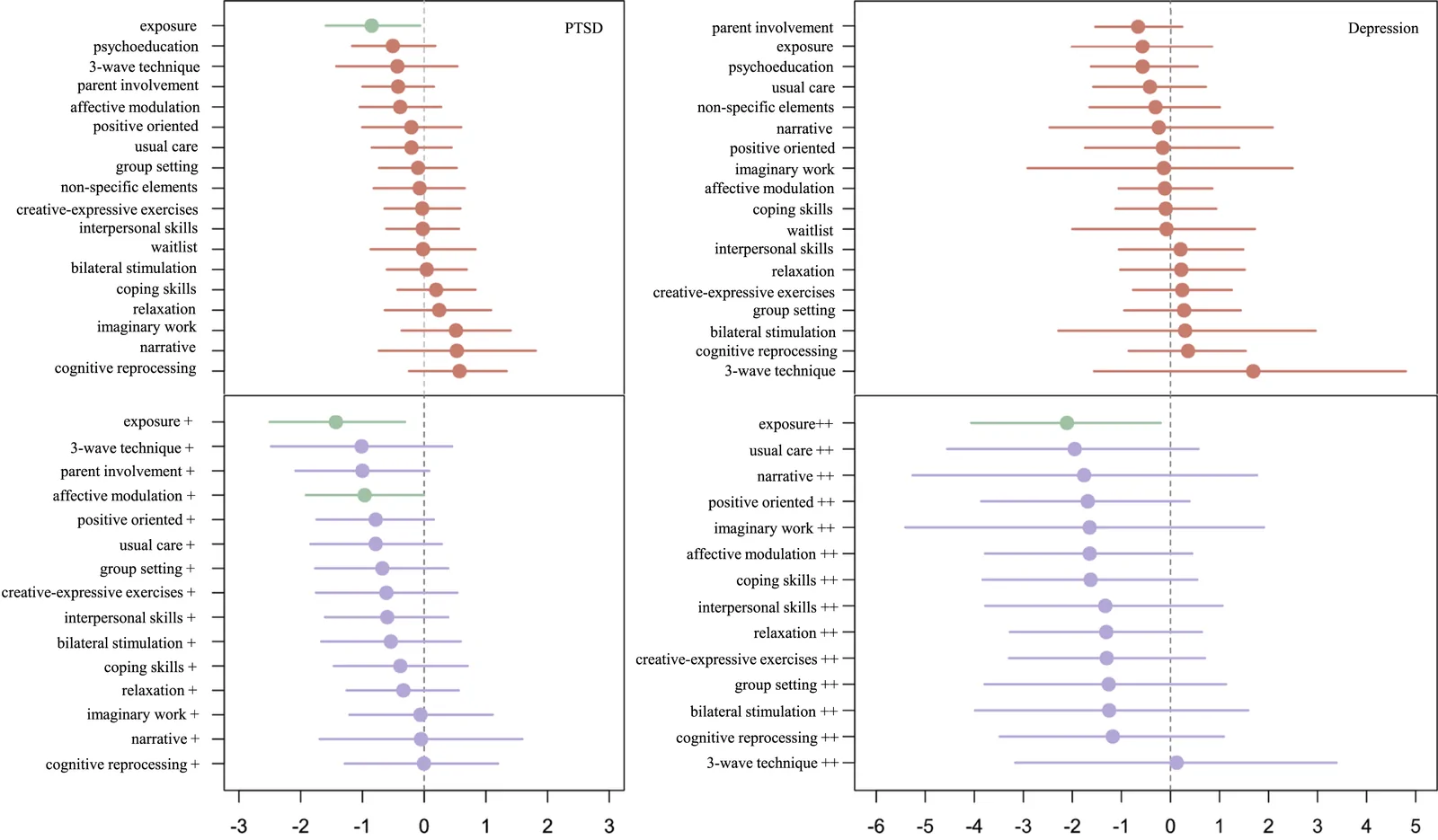
Forest plot of component effectiveness for PTSD and depression under individual and combined conditions. Each line in the figure represents the standardized mean difference (SMD) and 95% credible interval (CrI) for the corresponding component. The green line indicates that the 95% credible interval lies entirely below zero. The figures on top with brown lines represent the effects of individual components, while the figures on the bottom with purple lines represent the effects of combined components. A single plus sign (+) denotes a combination with nonspecific treatment elements and psychoeducation; two plus signs (++) denote a combination with nonspecific treatment elements, psychoeducation, and parental involvement.

### Sensitivity Analyses

Two approaches were used in the sensitivity analysis. First, age, sex, and pre-symptom severity were included as covariates in CNMA. Exposure remained the only individual component significantly associated with PTSD (SMD −1.00 [95% CrI −1.80 to −0.20]), and no individual component showed a significant effect on depression, consistent with the model without covariates (online suppl. material 15). Second, after excluding studies at high risk of bias, the posterior distributions widened, and no individual component remained statistically significant for PTSD. However, exposure retained a relatively high median effect size and a comparatively narrower posterior distribution. When examining the most parsimonious combinations, exposure and affective modulation demonstrated significant effects, each with the respective addition of parental involvement and a common therapeutic foundation (online suppl. material 15). Too few studies remained after excluding those at high risk of bias to perform a CNMA for depression.

## Discussion

This study is the first comprehensive meta-analysis to identify both effective interventions and their core therapeutic components for pediatric PTSD following complex trauma. By delineating individual therapeutic elements and the minimal effective combinations, we provide evidence-based guidance for scalable, resource-efficient solutions in response to rising global youth trauma exposure. Psychological interventions significantly reduced PTSD, depression, and anxiety symptoms. However, relative to passive control conditions, the effect size for PTSD was smaller (d = −0.77) than previously reported for single- or multiple-trauma PTSD (g = −1.09 to −1.11) [48]. This difference likely reflects our more rigorous trauma type-based definition of complex trauma rather than simple trauma counts. Our effect size suggests that complex trauma presentations may require more augmented and targeted approaches. Notably, psychological interventions were well tolerated, with a pooled dropout rate of 8%, consistent with prior meta-analytic estimates (11.7%) from general adolescent PTSD trials [49]. This slightly lower rate may be attributed to the higher proportion of group-based interventions in low-resource settings, which have been associated with improved retention [49]. No significant effects were found for dissociation or behavioral problems, despite their high prevalence in this population [50], aligning with prior reviews showing TF-CBT and EMDR provide no added benefit over usual care for behavioral outcomes when trauma type is unspecified [51]. These behavioral difficulties likely indicate multifaceted factors requiring additional support, such as stress regulation, impulse control, and family dynamics more explicitly [51]. This represents a critical treatment gap demanding innovative solutions.

Our analysis reveals concerns in the current evidence base. Selective reporting and incomplete outcome data were frequent sources of bias, though sensitivity analyses generally supported the robustness of our findings. Future studies should prioritize methodological rigor, transparency, and preregistration. The majority (87.5%) of trials focused exclusively on cognitive-behavioral approaches, reflecting CBT’s dominance while indicating limited exploration of alternative therapeutic modalities. We also identified notable research design disparities across regions: 75% of studies from low-resource settings used waitlist controls, compared to 36% in high-resource settings. Such asymmetries risk reinforcing inequities in guideline development by privileging evidence from high-resource contexts and undervaluing data from regions with the highest mental health needs, potentially limiting guideline relevance across diverse service contexts.

NMA demonstrated that trauma-focused treatments, including TF-CBT (across formats) and EMDR, were effective for PTSD, with TF-CBT also improving depression. These findings align with frontline treatment recommendations for adults with complex trauma [52, 53] and extend their relevance to younger populations. Notably, we found no significant differences in effectiveness or retention between phase-based and direct TF-CBT formats, consistent with recent RCTs directly comparing these approaches [22–25]. While phase-based treatment models have been widely advocated for PTSD associated with complex trauma exposure [21, 54], our results suggest that direct trauma processing is generally well tolerated in adolescents typically included in clinical trials. However, these trials often exclude participants with severe presentations (e.g., high suicidality or dissociative identity disorder), who may benefit from phased approaches [55]. The necessity to use phased versus direct treatment may depend not only on trauma type or PTSD severity but also on individual clinical characteristics. Future research should identify these specific factors to support more personalized intervention planning. The significant effects of both EMDR and MGI (integrating EMDR with non-TF-CBT techniques) in reducing PTSD align with reviews that do not differentiate trauma types in youth [51]. Preliminary case evidence also suggests potential benefit for ICD-11-defined CPTSD in adolescents [56]. Its nonverbal, sensory-based elements may be helpful for those who struggle to verbalize trauma, though empirical support remains limited. Additionally, child-centered play therapy showed large but marginally significant effects, likely due to only one eligible trial. Given its developmental appropriateness, especially for younger children, this modality warrants further investigation.

CNMA identified exposure as the only individually effective component for reducing PTSD symptoms, reinforcing its central role in trauma-focused therapies. Notably, previous studies have reported attenuated effects of exposure-based treatments in complex trauma populations [16]. One interpretation, drawing on Dalgleish and Power’s [57] framework, is that exposure effectively targets the structural pattern of re-experiencing and avoidance common across trauma responses, but may less directly address the diverse emotional appraisals – such as shame, guilt, or loss – often prominent in complex trauma. Future integrative approaches may retain exposure as a foundational element while incorporating additional components to address these broader emotional processes [58].

In addition to exposure alone, our analysis of minimally effective combinations revealed that affective modulation paired with foundational elements (i.e., nonspecific therapeutic factors and psychoeducation) was effective for PTSD symptom reduction. Affective modulation directly addresses emotional dysregulation, a core feature in complex trauma presentations, and may confer benefits across multiple symptom domains when combined with foundational support [59]. For depression outcomes, the most effective minimal combination included parental involvement, exposure, and foundational elements. Consistent with this, the NMA showed that non-trauma-focused CBT was associated with improved outcomes when parental involvement was included. These findings align with research highlighting the importance of caregiver support in adolescent trauma recovery [60, 61]. Prior meta-analyses found that parental involvement contributed to recovery among sexually abused children [62] but showed no significant influence on treatment outcomes in studies that did not differentiate trauma type [35, 63]. Taken together, these findings suggest that parental involvement may have modest but meaningful effects, particularly in youth exposed to complex trauma, though differences in study design may partly account for these patterns.

Our CNMA findings indicate that specific component combinations are necessary for addressing complex trauma presentations [64], yet the relationship between treatment complexity and outcomes appears nonlinear. Excessive complexity may dilute clinical focus, reduce implementation fidelity, or compromise feasibility, especially in resource-limited settings. Identifying essential component combinations is particularly relevant in low-resource contexts, where streamlined interventions that maintain therapeutic effectiveness could significantly improve treatment accessibility, scalability, and sustainable implementation.

Several limitations warrant acknowledgment. First, this meta-analysis was restricted to studies in which PTSD symptoms were the primary outcome. Although comorbidity data were extracted when available, limited reporting precluded comprehensive coverage of the broad mental health sequelae associated with complex trauma exposure. Second, inclusion was based on trauma exposure histories rather than formal ICD-11 diagnostic classifications; findings should therefore not be interpreted as specific to CPTSD. Third, because our analyses relied on aggregate study-level data, associations with individual-level characteristics (e.g., age, sex) are subject to ecological bias. Associations observed across studies may reflect between-study differences rather than within-study or individual-level relationships and should not be interpreted as evidence of individual-level effect modification [65, 66]. Fourth, the CNMA relied on binary component coding and therefore cannot capture variation in component operationalization, including the amount or intensity with which each component was delivered. Such variation may arise from differences in treatment duration, component sequencing, and implementation fidelity across studies, which may influence effect estimates and limit generalizability.

## Conclusions

To conclude, this comprehensive multitiered meta-analysis reveals which treatments, and which specific components, effectively address trauma-related symptoms in youth with complex trauma histories. Trauma-focused treatments demonstrated broad effectiveness, with exposure, parental involvement, and affective components emerging as crucial therapeutic elements. Notably, phase-based approaches conferred no additional benefit over direct trauma-focused approaches in typical trial populations. Given the global need for accessible trauma care, our results support task-shifting models by identifying essential components suitable for nonspecialist delivery, potentially expanding access without compromising effectiveness. This evidence offers a foundation for developing context-sensitive, developmentally appropriate clinical guidelines for complex trauma care in diverse settings.

## Statement of Ethics

An ethics statement is not applicable because this study is based exclusively on published literature.

## Conflict of Interest Statement

The authors have no conflicts of interest to declare.

## Funding Sources

This work was supported by the National Natural Science Foundation of China (Grant Nos. 32371139, 32000776), the Open Funding of the National Key Laboratory of Cognitive Neuroscience and Learning (Grant No. CNLZD2103), and the the Henry Fok Macau Foundation (Fok Ying Tung Foundation). The funders had no role in the study design, execution and analysis, and manuscript conception, planning, writing, and decision to publish.

## Author Contributions

Y.Z., Y.L., and J.F. contributed to the conceptualization of the study. Y.L. and J.F. registered the protocol, performed data analysis, and drafted the initial manuscript. Y.L., W.H., Q.P., Z.Z., and J.W. conducted the literature search and data extraction. Y.L. and J.W. assessed the risk of bias. W.H. and M.L. verified the data and contributed to manuscript preparation. Y.Z., T.E., and A.K. critically reviewed and revised the final version of the manuscript.

## Data Availability

All data generated or analyzed during this study are included in this article and its online supplementary material. Further inquiries can be directed to the corresponding author.
